# Impact of a varied set of stimuli on a suite of immunological parameters within peripheral blood mononuclear cells: toward a non-animal approach for assessing immune modulation by materials intended for human use

**DOI:** 10.3389/ftox.2024.1335110

**Published:** 2024-04-26

**Authors:** Stella Cochrane, Ramya Rajagopal, David Sheffield, Fay Stewart, Lindsay Hathaway, Nicholas M. Barnes, Omar Qureshi, John Gordon

**Affiliations:** ^1^ Safety and Environmental Assurance Centre (SEAC), Unilever, Colworth Science Park, Sharnbrook, United Kingdom; ^2^ Celentyx Ltd., Birmingham Research Park, Birmingham, United Kingdom; ^3^ Institute for Clinical Sciences, University of Birmingham, Birmingham, United Kingdom

**Keywords:** peripheral blood mononuclear cell, immune modulation, *in vitro*, non-animal, toxicology, T cell, B cell, monocyte

## Abstract

**Introduction:** In toxicology, steps are being taken towards more mechanism-focused and human relevant approaches to risk assessment, requiring new approaches and methods. Additionally, there is increasing emphasis by regulators on risk assessment of immunotoxicity.

**Methods:** Here we present data from a peripheral blood mononuclear cell (PBMC) system whereby a varied set of stimuli, including those against the TCR and Toll-like receptors, enable readouts of cytokine and prostaglandin E2 (PGE2) production with monocyte, T cell and B cell viability, proliferation, and associated activation markers. In addition to results on the impact of the stimuli used, initial profiling data for a case study chemical, curcumin, is presented, illustrating how the system can be used to generate information on the impact of exogenous materials on three major constituent immune cell subsets for use in risk assessment and to direct follow-on studies.

**Results:** The different stimuli drove distinct responses, not only in relation to the “quantity” of the response but also the “quality”. Curcumin had a limited impact on the B cell parameters measured, with the stimuli used, and it was noted that in contrast to T cells where there was either no impact or a reduction in viability and proliferation with increasing concentration, for B cells there was a small but significant increase in both measurements at curcumin concentrations below 20 µM. Similarly, whilst expression of activation markers by T cells was reduced by the highest concentration of curcumin, they were increased in B cells. Curcumin only impacted the viability of stimulated monocytes at the highest concentration and had differential impact on different activation markers. Levels of all cytokines and PGE2 were reduced at higher concentrations.

**Discussion:** Although the platform has certain limitations, it nevertheless enables assessment of healthy baseline monocyte, T-, and B-cell responses, and scrutiny of the impact of different stimuli to detect potential immune suppression or enhancement from exogenous materials. In the case of curcumin, a pattern of responses indicative of immune suppressive / anti-inflammatory effects was detected. It is an accessible, highly modifiable system that can be used to screen materials and guide further studies, providing a holistic, integrated picture of effects.

## 1 Introduction

Understanding the impact of different materials on immune cell subsets is an important aspect of the efficacy and safety assessment of materials intended for human use as a part of an overall testing strategy to meet regulatory requirements and ensure the protection of human health. There is also increasing interest in doing this without the use of animals (Dent et al., 2021; Naidenko et al., 2021; Wang et al., 2022). In the field of toxicology, steps are being taken toward more mechanism-focused and human-relevant approaches to risk assessment, driving the development of new methods, including *in vitro* methods, to assess the effects of materials on the human immune system. More recently, there has been increasing emphasis by regulators and the wider scientific community on toxicity associated with immune responses (Hofer et al., 2022; EPRS, 2023). It is recognized that the complexity of the immune system and the need to cover all “arms,” i.e., innate, adaptative, and subsets thereof, are such that no single test will be able to detect all types of immune modulation, but rather, a range of approaches and assays will be required that can be applied in a tiered manner (Hartung and Corsini, 2013; Karmaus and Karmaus, 2018; Maddalon et al., 2023). Here, we present data from a human peripheral blood mononuclear cell (PBMC) system, where a varied set of stimuli enables readouts of cytokine production, monocyte, T-cell, and B-cell proliferation and viability, and T-cell, B-cell, and monocyte activation markers in cultures supported by human serum rather than fetal calf serum. This human-focused platform thus provides insights into how three major constituent immune cell subsets from different arms of the human immune system are impacted by different treatments. The stimuli selected for investigating the effects on T cells and expected to activate the TCR were CytoStim (CS), tetanus toxin (TT), and house dust mite (HDM) extract. CS is an antibody-based synthetic superantigen that activates T cells by cross-linking the TCR with MHCII. Superantigens normally do not require processing and act as a strong polyclonal or nonantigen-specific stimulus that is often independent of co-stimulation (Damle et al., 1993; Campbell et al., 2011). TT stimulates a specific clonal T-cell response after undergoing processing (Demotz et al., 1989; Adams et al., 1991). Proteins from house dust mites cause IgE-mediated allergic sensitization, which requires helper T (type 2) cells (Jacquet, 2013) and would be expected to require antigen processing to elicit responses from antigen-specific T-cell populations. In addition, other proteins in the preparation may trigger innate cell activation. The two stimuli selected for investigating B-cell readouts were chosen to cover T-cell-dependent [pokeweed mitogen (PWM)] and T-cell-independent mechanisms [TLR9 activation by CpG ODN (CpG) with support from IL-15] (Gmelig-Meyling et al., 1977; Huggins et al., 2007). Pokeweed mitogen acts on both B cells and T cells and can drive T-cell-dependent B-cell activation. CpG ODN triggers TLR9 activation and, in combination with IL-15, provides a strong B-cell stimulus. In addition, CpG ODN may also activate TLR9-expressing innate cell types within PBMCs, such as monocytes and plasmacytoid dendritic cells (DCs) (Horii and Hhirano, 1998; Bauer et al., 1999; Moseman et al., 2004; Mauri et al., 2014; Gupta et al., 2018). For investigating the effects on monocytes, peptidoglycan (Pep), the principal component of the Gram-positive bacterial cell wall, and lipopolysaccharide (LPS) from Gram-negative bacteria were utilized. Pep would lead to the activation of NOD-like receptors (NOD1 and NOD2) and, in isolation, in PBMC cultures, would be expected to activate monocytes, although it may also sensitize CD8 T cells to TCR signals and activate NK and NKT cells (Mercier et al., 2012; Selvanantham et al., 2013). LPS would drive TLR4 activation on monocytes and other innate immune cells. Our results reveal differences in both the quality and the magnitude of the responses elicited by these varied stimuli, as well as a range of healthy donor responses. To illustrate how the system can be used for an *ab initio* assessment of the impact of test materials on three major immune cell subsets, we present an initial profiling of the impact of curcumin within this system as a case study material with reported anti-inflammatory properties and discuss the implications for application in risk assessment. Curcumin is one of a range of materials with reported immunomodulatory activity in humans, selected based on information indicating that they act via different mechanisms and with different potencies, that has been assessed using the platform. Curcumin was specifically chosen as one of the test materials for assessment as it has reported anti-inflammatory properties, although this effect is surrounded by a degree of controversy (Nelson et al., 2017), as covered in the discussion.

## 2 Materials and methods

Peripheral blood mononuclear cells were isolated from eight healthy donors through Ficoll-Paque PLUS (GE Healthcare; 11778538) density centrifugation. All samples were obtained with informed consent and approval from the London–South East Research Ethics Committee (REC reference: 16/LO/0601).

Curcumin (Sigma-Aldrich, C1386) was reconstituted in DMSO (Sigma-Aldrich) at concentrations of 0.078, 0.3125, 1.25, 5.0, and 20 μM and stored at −20°C. These concentrations were selected after reviewing the available literature on effects in other *in vitro* assays and cell lines, including cytotoxicity and potential human serum levels from oral exposure. Stimulation reagents were reconstituted in HyClone HyPure WFI Quality Water (GE LifeSciences) at the concentrations indicated (Table 1), which were selected based on available information regarding the impact on PBMCs and supplier recommendations with the aim of providing “high, medium, and low” levels of stimulation and stored at −20°C, except as indicated below. The reagents used were as follows:

• For T-cell assays: tetanus toxin (Sigma-Aldrich, T3194), house dust mite antigen (*Dermatophagoides pteronyssinus* extract; Citeq Biologics, product code 02.01.85), and CytoStim (Miltenyi Biotec, 130-092-173) were stored at 4°C.
• For B-cell assays: CpG ODN (InvivoGen, tlrl-2006), IL-15 (ImmunoTools, 11340157), and pokeweed mitogen (Sigma-Aldrich, L8777), which was reconstituted in PBS.
• For monocyte assays: peptidoglycan (Sigma-Aldrich, SMB0028) and LPS (InvivoGen, tlrl-b5lps).

**TABLE 1:** Final and stock concentrations of stimulation reagents.

Stimulation reagent	Stock concentration	Final concentration
CytoStim	n/a	1.2, 5.0, and 20 μL/mL
Tetanus toxin	250 μg/mL in water	250, 500, and 1,000 ng/mL
House dust mite antigen	1.0 mg/mL in water	2.0, 10, and 50 μg/mL
CpG ODN + IL-15	CpG ODN: 500 µM in water IL-15: 100 μg/mL in water	0.04, 0.2, and 1.0 µM CpG ODN + 15 ng/mL IL-15
Pokeweed mitogen	1 mg/mL in water	1.0, 5.0, and 10 μg/mL
Peptidoglycan	10 mg/mL in water	10, 100, and 1,000 ng/mL
LPS	5 mg/mL in water	0.01, 1.0, and 100 ng/mL

PBMCs were isolated and used fresh, cultured at 2 × 10^5^ cells/well in 0.1% DMSO or with curcumin for 1 h and then cultured in the absence (unstimulated) or presence of stimulating reagents at the concentrations given in Table 1.

Cultures were set up in RPMI 1640 Medium (with sodium bicarbonate and L-glutamine; Sigma-Aldrich) with 1% (v/v) penicillin/streptomycin (Sigma-Aldrich) and 5% (v/v) heat-inactivated human serum (Tissue Solutions Ltd.) in 96-well round bottom plates (Sarstedt) for 24 h (monocyte cultures) or 6 days (T- and B-cell cultures) at 37°C, 5% CO_2_.

After 24 h (monocyte assays) or 6 days (T- and B-cell assays) of cell culture, the plates were centrifuged to pellet cells, and supernatants were removed and stored at −20°C for the subsequent analysis of soluble readouts.

The cells were then stained as follows:

• For T-cell assays: anti-human CD3 PerCp/Cy5.5, CD4 PE, CD8 FITC, CD25 APC, and CD71 Pe/Cy7 (all BioLegend; 317336, 317410, 301050, 302610, and 334112, respectively), as well as Zombie NIR viability dye (BioLegend; 423106), followed by fixation using the FoxP3 Staining Buffer Set (BD Biosciences; 560098).
• For B-cell assays: anti-human CD19 PerCp/Cy5.5, CD71 Pe/Cy7, HLA-DR Alexa Fluor 488, and CD86 PE (all BioLegend; 363016, 334112, 338514, 307620, and 374206, respectively), as well as Zombie NIR viability dye (BioLegend; 423106), followed by fixation using the FoxP3 Staining Buffer Set (BD Biosciences; 560098)
• For monocyte assays: anti-human CD14 Pe/Cy7, CD56 Brilliant Violet 421, CD3 PerCp/Cy5.5, HLA-DR Alexa Fluor 488, CD86 PE, CD25 APC, and CD69 Brilliant Violet 510 (all BioLegend; 325618, 318328, 317336, 307620, 374206, 302610, and 310936, respectively), as well as Zombie NIR viability dye (BioLegend; 423106), followed by fixation using the FoxP3 Staining Buffer Set (BD Biosciences, 560098).

Following fixation, which was done by diluting the fix concentrate 10-fold in distilled water and adding 100 μL of fix buffer per well, incubation for 10 min at room temperature was performed in the dark. The cells were then washed and resuspended in FACS buffer (PBS + 0.2% heat-inactivated human serum). AccuCheck Counting Beads (Thermo Fisher, PCB100) were added to each sample immediately before analysis by flow cytometry. The results were analyzed using FlowJo 10.3. The gating for T cells, B cells, and monocytes was as follows: FSc vs SSc (Cells) >PulseWidth vs FSc (singlets)> viability vs SSc (live cells)> CD3 for T cells plus CD4 or CD8, CD19 for B cells, and CD14 for monocytes vs SSc.

The following cytokines were measured in the cell culture supernatants using Luminex (Bio-Rad) following the manufacturer’s instructions and using the Bio-Plex 200 system and Bio-Plex Manager software.

• For T-cell assays: IL-2, IL-6, IL-8, IL-10, IL-17A, IFNγ, and TNFα
• For B-cell assays: IL-2, IL-4, TNFα, IFNγ, IL-12, and IL-17A
• For monocyte assays: IL-1β, TNFα, IL-6, IL-8, IL-10, IL-12, IL-15, and IL-18. Additionally, levels of prostaglandin E2 (PGE2) were measured in these supernatants using the PGE2 ELISA Kit (Enzo Life Sciences, ADI-900-001) following the manufacturer’s instructions.

For each donor, isolated PBMCs were plated out in triplicate for each treatment condition (e.g., unstimulated, stimulation with 1.2 µL CS, or stimulation with 1.2 µL CS, followed by treatment with curcumin), providing three replicate measurements for each parameter for each treatment condition per donor.

### 2.1 Statistics

To compare the results from each stimulation level against the unstimulated results, a model was fit to the logged values using the MIXED Procedure in SAS 9.4 (SAS Institute). Individual donor effects were accounted for using fixed effects, and the stimulation levels were compared using estimate statements. Statistical tests were at the 5% confidence level.

## 3 Results

### 3.1 T cells

#### 3.1.1 Impact of CS, TT, and HDM on T-cell viability and proliferation

**FIGURE 1:**
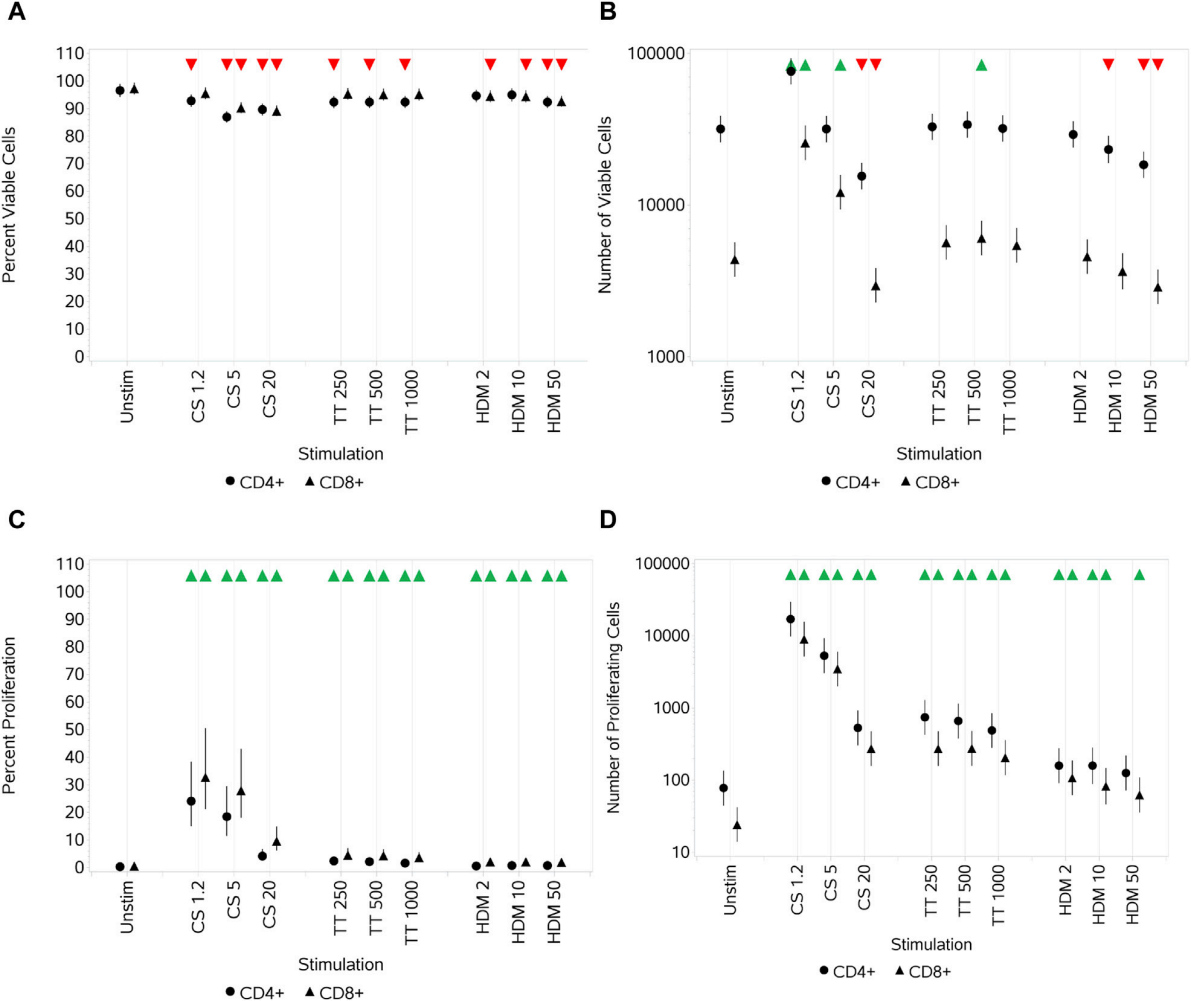
Impact of stimuli on CD4 and CD8 T-cell viability and proliferation measured as the percentage viable cells **(A)**, absolute number of viable cells **(B)**, percentage of proliferating cells **(C)**, and number of proliferating cells **(D)**. Red triangles denote a significant decrease compared to the unstimulated control, and green triangles indicate a significant increase. Points and lines represent the mean and 95% confidence interval of the mean after the donor effect has been accounted for (eight donors, each with three measurements), respectively.

A relatively small but significant decrease in the percentage of viable CD4^+^ and CD8^+^ T cells was observed with all three stimuli when compared to unstimulated cultures (Figure 1A). This effect on viability was observed at all concentrations of CS on CD4^+^ T cells but only at the medium and high concentrations of CS on CD8^+^ T cells. All concentrations of TT resulted in a decrease in the percent viability of CD4^+^ but not CD8^+^ T-cell populations. In contrast, the HDM stimulus led to a decrease in the percent viability of CD8^+^ T cells at all concentrations but only at its highest for CD4^+^ T cells. In all cases, the % viability of the cells was above 80%. When observing the total number of viable CD4^+^ and CD8^+^ T cells, an increase in both subsets at the lowest concentration of CS and in CD8^+^ cells at the medium concentration was observed. At the highest CS concentration, the numbers of both subsets were significantly reduced compared to the control (Figure 1B). With TT as the stimulus, the only significant change in the number of viable cells observed was a small increase in CD8^+^ T cells with the medium concentration. With HDM, the lowest concentration had no significant impact, the medium concentration reduced the number of CD4^+^ but not CD8^+^ T cells, and the highest concentration reduced the number of both subsets. All three stimuli, at all concentrations, resulted in a significant (although relatively small in the cases of TT and HDM) increase in the percentage of proliferating CD4^+^ and CD8^+^ T cells compared to unstimulated cells (Figure 1C). Each of the stimuli also led to an increase in the total number of proliferating cells at all concentrations, except for CD4^+^ T cells at the highest HDM concentration (Figure 1D). CS had the greatest impact on proliferation compared to TT or HDM, and the greatest increases were observed at its lowest concentrations (Figures 1C, D).

#### 3.1.2 Impact of CS, TT, and HDM on T-cell activation markers CD25 and CD71

**FIGURE 2:**
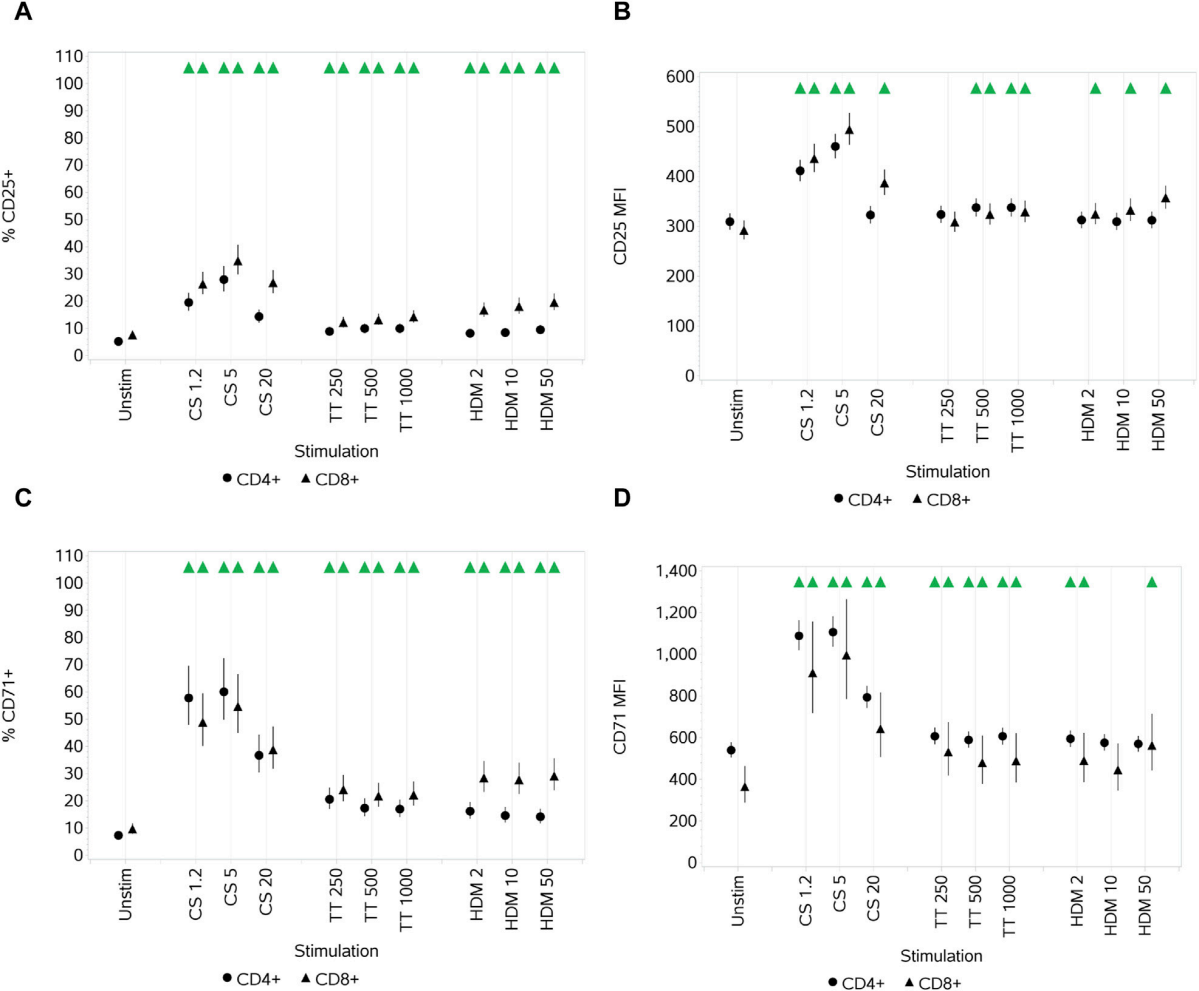
Impact of stimuli on the activation markers CD25 and CD71 on CD4 and CD8 T cells measured as % CD25^+^ cells **(A)**, CD25 median fluorescence intensity (MFI) **(B)**, % CD71^+^ cells **(C),** and CD71 MFI **(D)**. Red triangles denote a significant decrease compared to the unstimulated control, and green triangles indicate a significant increase. Points and lines represent the mean and 95% confidence interval of the mean after the donor effect has been accounted for (eight donors, each with three measurements), respectively.

Each of the stimuli drove a significant increase in the percentage of cells that expressed CD25 compared to unstimulated cells (although a relatively small increase (especially for TT and HDM)) at all three concentrations (Figure 2A). For CS, this peaked with the medium concentration. An increase in CD25 median fluorescence intensity (MFI) was also observed at the low and medium CS concentrations (highest with the medium), decreasing such that the MFI was still higher than that for untreated CD8^+^ T cells at the highest concentration but not for CD4^+^ T cells. A smaller increase in the MFI was observed with both cell types with the medium and high TT concentrations, but only CD8^+^ T cells had a small increase in the MFI with HDM across all concentrations (Figure 2B).

All stimuli resulted in a significant increase, at all three concentrations, in the percentage of CD4 and CD8^+^ T cells that expressed CD71 compared to unstimulated cells (Figure 2C). CS and TT also led to an increase in the CD71 MFI of both CD4^+^ and CD8^+^ T cells across all concentrations (Figure 2D). HDM resulted in a significant increase in CD71 MFI for both T-cell subsets only at the low concentration, but for CD4^+^ cells, the increase was also noted at the high concentration. For the two subsets, with respect to both % activation and the MFI, CS had a greater impact than TT and HDM, and the greatest impact was with the low and medium CS concentrations, decreasing at the highest concentration.

#### 3.1.3 Impact of CS, TT, and HDM on cytokine production

**FIGURE 3:**
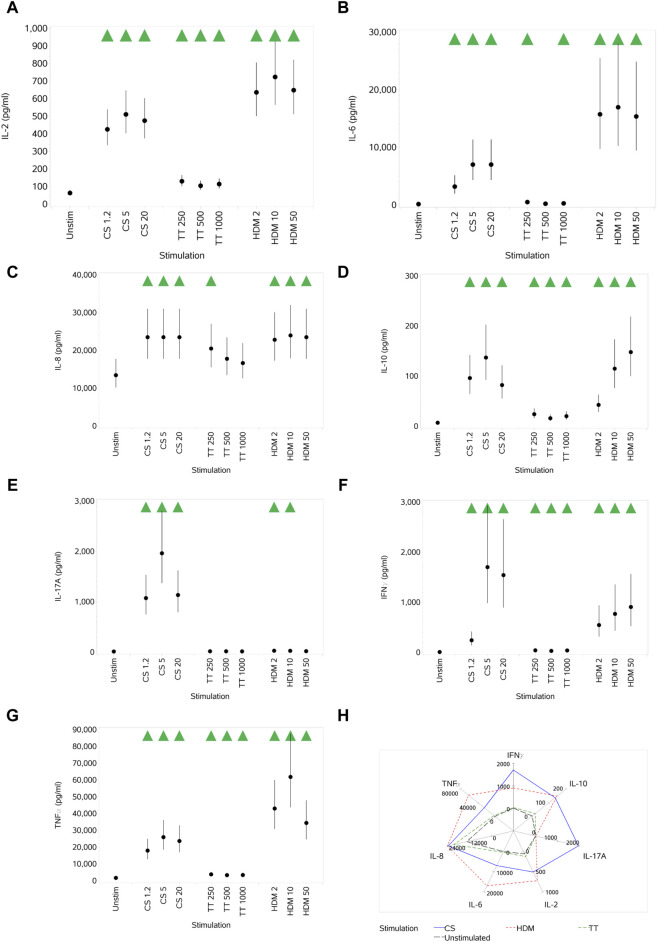
Impact of stimuli on the levels of a range of cytokines: IL-2 **(A)**, IL-6 **(B)**, IL-8 **(C)**, IL-10 **(D)**, IL-17A **(E)**, IFNγ **(F)**, and TNFα **(G)**. Spider plot capturing the pattern of expression for the different stimuli **(H)**. Red triangles denote a significant decrease compared to the unstimulated control, and green triangles indicate a significant increase. Points and lines represent the mean and 95% confidence interval of the mean after the donor effect has been accounted for (eight donors, each with three measurements), respectively.

CS stimulation increased the secretion of all cytokines (IL-2, IL-6, IL-8, IL-10, IL-17A, IFNγ, and TNFα) at all concentrations, with peak secretion levels at the medium concentration (Figures 3A–G). TT, relative to CS and HDM, was a weak cytokine inducer, failing to induce any increase in IL-17A levels and only small increases in others and not at all concentrations. HDM also elicited an increased secretion of all cytokines at all concentrations, although the impact on IL-17A was weak. In some cases, the impact of HDM was greater than that of CS, while, in others, it was weaker. The differential effects of the different stimuli are shown in Figure 3H, where the maximal responses for each are plotted. CS does not have as high an effect on TNFα levels as HDM, whereas HDM does not affect the IL-17A levels as much as the CS stimulus. Other than IL-8, the cytokine responses elicited by TT were of lower magnitude than the other two stimuli.

### 3.2 B cells

#### 3.2.1 Impact of PWM and CpG on B-cell viability and proliferation

**FIGURE 4:**
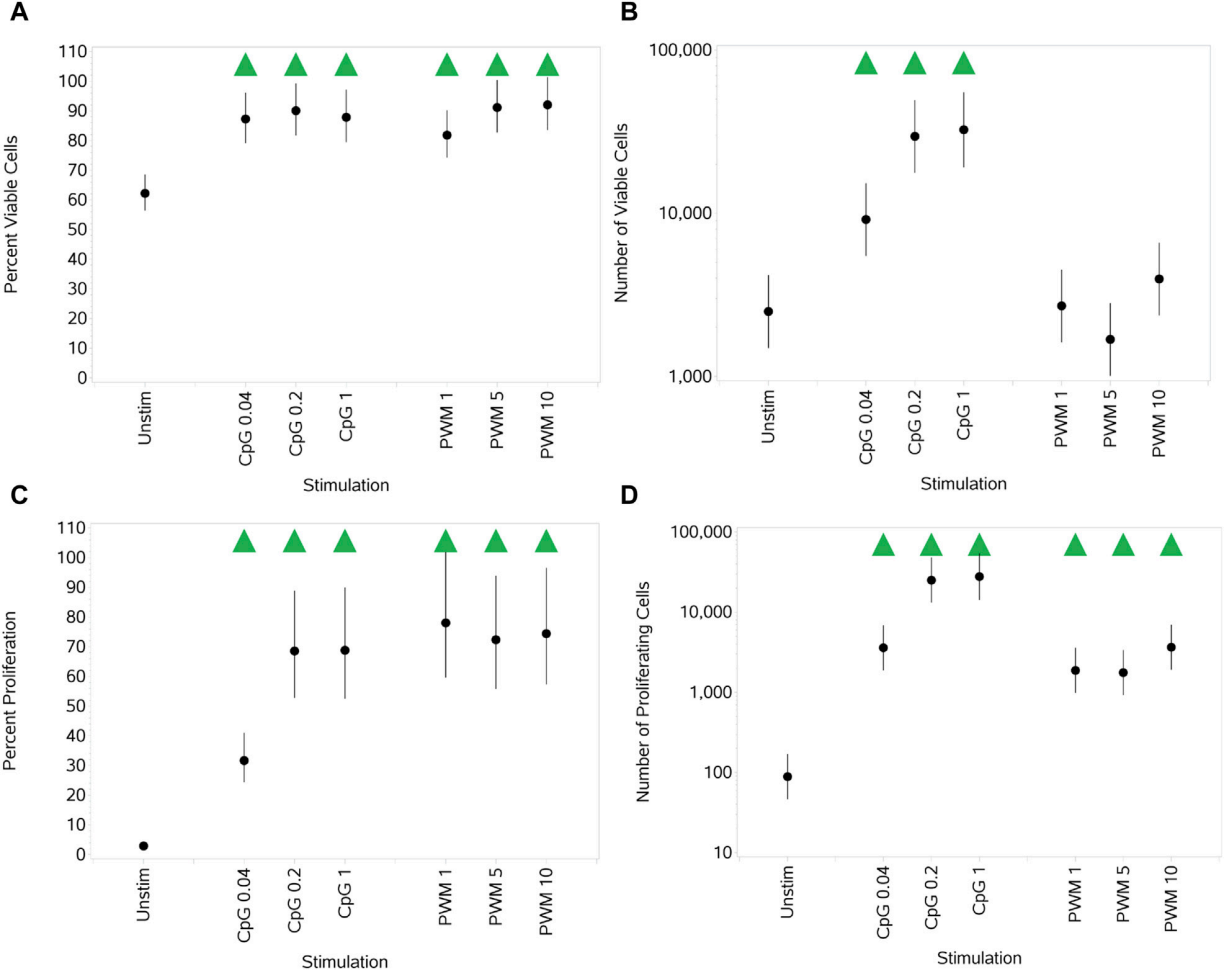
Impact of stimuli on B-cell viability and proliferation measured as the percentage viable cells, **(A)** absolute number of viable cells **(B)**, percentage of proliferating cells **(C)**, and number of proliferating cells **(D)**. Red triangles denote a significant decrease compared to the unstimulated control, and green triangles indicate a significant increase. Points and lines represent the mean and 95% confidence interval of the mean after the donor effect has been accounted for (eight donors, each with three measurements), respectively.

In contrast to the results observed for T cells, a significant increase in the percentage of viable B cells was observed with the two stimuli when compared to unstimulated cells (Figure 4A). This effect was observed at all concentrations. When observing the total number of viable B cells (Figure 4B), an increase with increasing concentrations of CpG that started to plateau at the top two concentrations was observed. In contrast, with PWM, no increase was observed in the number of viable B cells compared to unstimulated cultures. Both stimuli, at all concentrations, resulted in a significant increase in the percentage of proliferating B cells compared to unstimulated cultures. With CpG, this increase started to plateau at the two highest concentrations (Figure 4C), while with PWM, all concentrations resulted in a similar increased percentage. The same pattern of response was observed when assessing the absolute number of proliferating B cells (Figure 4D).

#### 3.2.2 Impact of PWM and CpG on B-cell activation markers CD71, CD86, and HLA-DR

**FIGURE 5:**
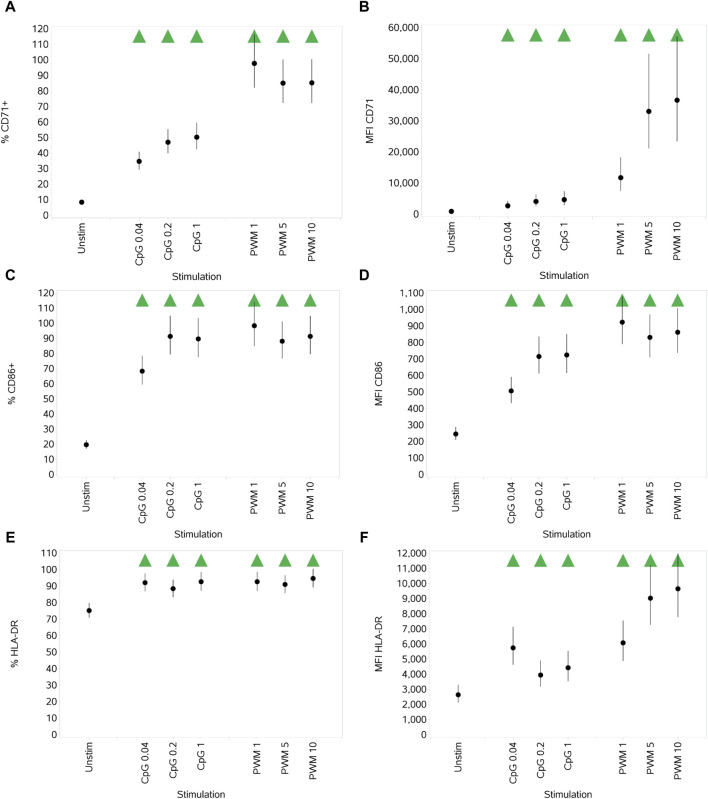
Impact of stimuli on the activation markers CD71, CD86, and HLA-DR on B cells measured as % CD71^+^ cells **(A)**, CD71 MFI **(B)**, % CD86^+^ cells **(C)**, CD86 MFI **(D)**, % HLA-DR^+^ cells **(E)**, and HLA-DR MFI **(F)**. Points and lines represent the mean and 95% confidence interval of the mean after the donor effect has been accounted for (eight donors, each with three measurements), respectively.

Both stimuli significantly increased the expression of all three activation markers (both % activation and MFI) compared to unstimulated cells. Of note, PWM was generally the stronger stimulus of the two with respect to induced activation marker upregulation, which was particularly marked for CD71 (Figures 5A–F).

#### 3.2.3 Impact of PWM and CpG on cytokine production

The impact of stimuli on cytokine production (IL-2, IL-4, IL-17A, IFNγ, TNFα, and IL-12) was investigated, and while a strong cytokine response to stimulation with PWM was observed, it was very muted (changes were small/non-significant) for CpG, with a general pattern of a little suppression at the lowest dose and then no change/small increase at the two higher concentrations. PWM consistently led to significant, much larger increases (many fold higher than stimulated by CpG) in expression across all concentrations. As any changes in the cytokine expression for PWM may primarily be coming from activated T cells, the data are not included here.

### 3.3 Monocytes

#### 3.3.1 Impact of Pep and LPS on monocyte viability

**FIGURE 6:**
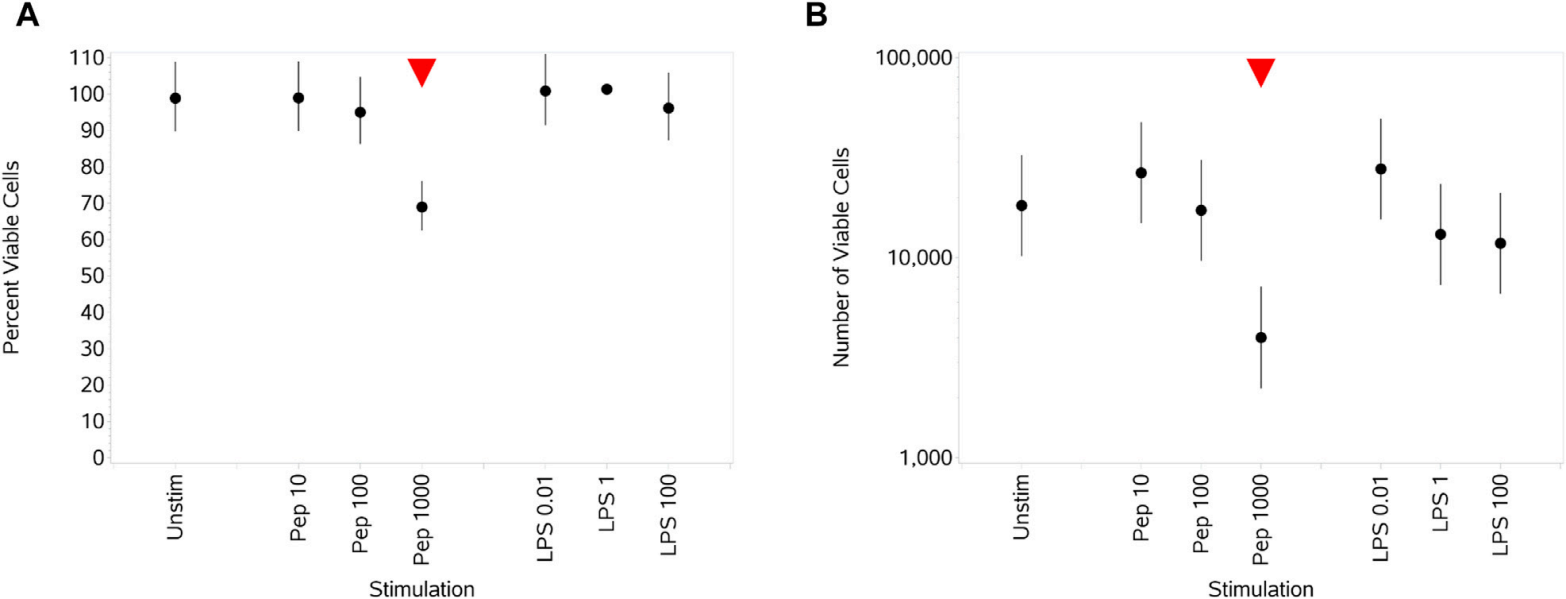
Impact of stimuli on monocyte viability measured as the percentage viable cells **(A)** and absolute number of viable cells **(B)**. Red triangles denote a significant decrease compared to the unstimulated control, and green triangles indicate a significant increase. Points and lines represent the mean and 95% confidence interval of the mean after the donor effect has been accounted for (eight donors, each with three measurements), respectively.

The only impact of the stimuli here was a significant decrease in both percentage and absolute numbers of viable monocytes (Figures 6A, B) at the highest concentration of Pep compared to unstimulated monocytes. Overall, high inter-individual variability between the donors in the number of viable cells led to large error bars for all concentrations of both stimuli (data not shown).

#### 3.3.2 Impact of Pep and LPS on monocyte activation markers CD25, CD86, and HLA-DR

**FIGURE 7:**
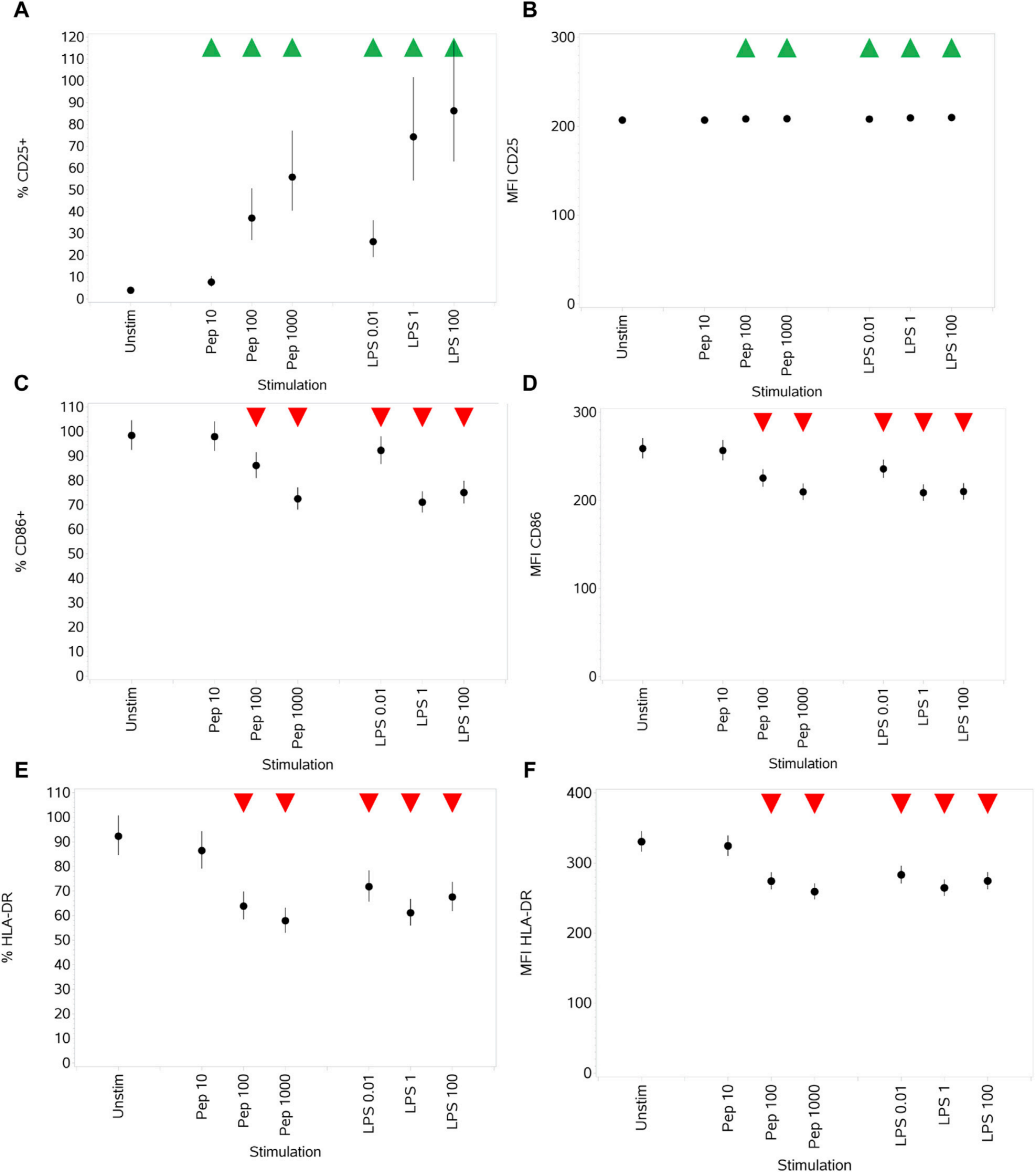
Impact of stimuli on the activation markers CD25, CD86, and HLA-DR measured as % CD25^+^ cells **(A)**, CD25 MFI **(B)**, % CD86^+^ cells **(C)**, CD86 MFI **(D)**, % HLA-DR^+^ cells, **(E)** and HLA-DR MFI **(F)**. Points and lines represent the mean and 95% confidence interval of the mean after the donor effect has been accounted for (eight donors, each with three measurements), respectively.

Both stimuli drove an increase in the percentage of monocytes expressing CD25, compared to unstimulated cells, in a concentration-dependent manner (Figure 7A), which was reflected by a small change in MFI values (Figure 7B). Medium and high concentrations of Pep and all concentrations of LPS drove a decrease in both the percentage of monocytes expressing CD86 and HLA-DR and the MFI values (Figures 7B–F).

#### 3.3.3 Impact of Pep and LPS on cytokine and PGE2 production

**FIGURE 8:**
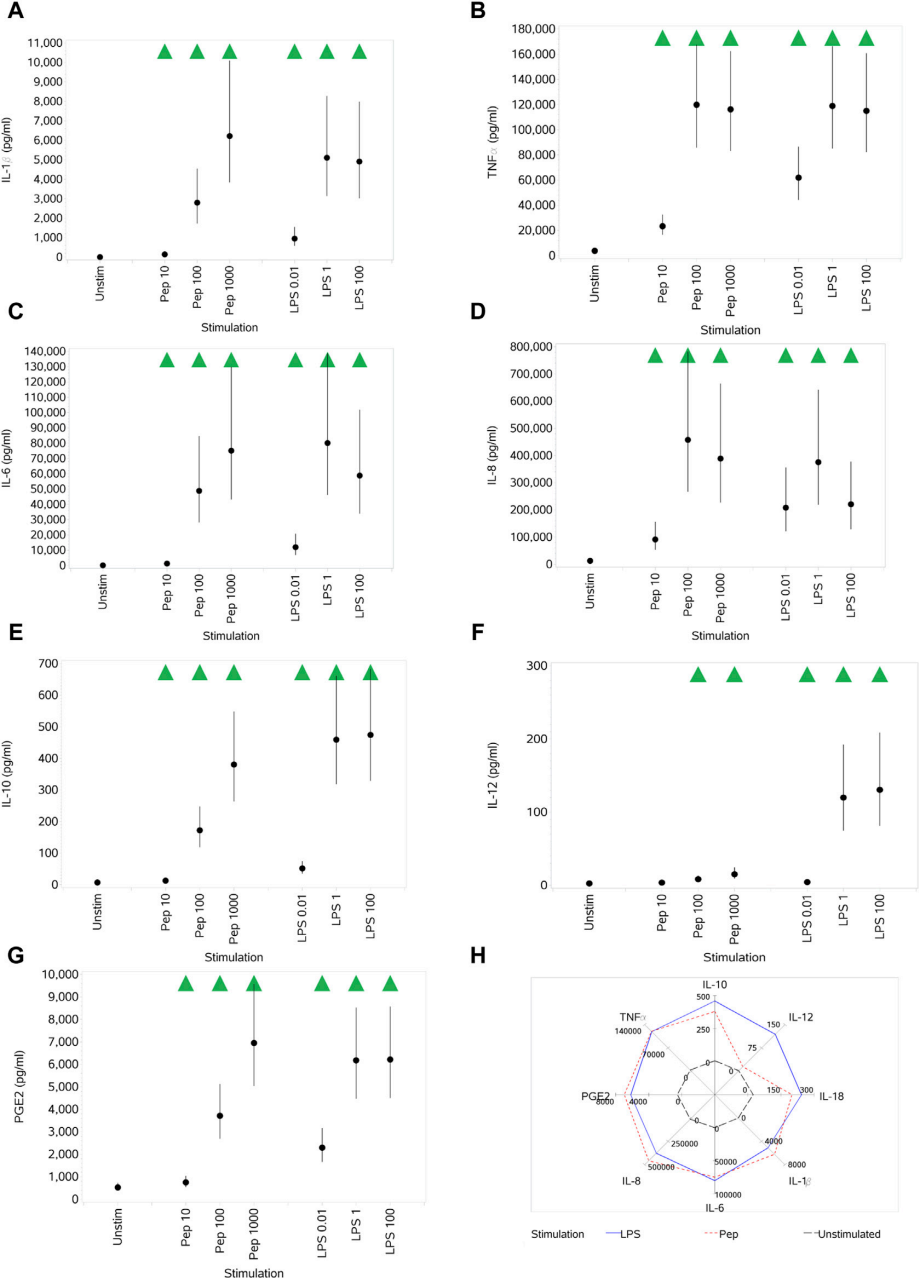
Impact of stimuli on levels of a range of cytokines and PGE2: IL-1β **(A)**, TNFα **(B)**, IL-6 **(C)**, IL-8 **(D)**, IL-10 **(E)**, IL-12 **(F)**, and PGE2 **(G)**. Spider plot capturing the pattern of expression for the different stimuli **(H)**. Red triangles denote a significant decrease compared to the unstimulated control, and green triangles indicate a significant increase. Points and lines represent the mean and 95% confidence interval of the mean after the donor effect has been accounted for (eight donors, each with three measurements), respectively.

Both Pep and LPS exposure resulted in the increased secretion of IL-1β, TNFα, IL-6, IL-8, IL-10, IL-12, IL-18, and PGE2 at almost all concentrations, noting, however, the different potencies of stimuli when comparing the different concentration ranges of each (10–1,000 ng/mL Pep versus 0.01–100 ng/mL LPS) (Figures 8A–H). A notable difference between the stimuli was the impact on IL-12, where only the medium and high concentrations of Pep resulted in a significant increase compared to unstimulated monocytes but with the increase being much lower than that stimulated by LPS (see Figures 8F, H). A figure is not provided for IL-18, but the pattern of response was the same as that for IL-1β (Figure 8A), although the levels of IL-18 produced were much lower (up to ∼ 3 pg/mL).

### 3.4 Impact of curcumin

#### 3.4.1 Impact of curcumin on T-cell viability and proliferation unstimulated and stimulated with CS or HDM

When unstimulated PBMCs (data not shown) were treated with a range of concentrations of curcumin (0.078 µM–20 µM), no significant impact on viability (% or absolute cell numbers) was observed other than a very small increase in the percentage of viable CD4^+^ T cells treated with 20 µM curcumin. Proliferation (both % and absolute cell numbers) of CD4^+^ T cells, noting that the control level was extremely low, was reduced by all concentrations. For CD8^+^ T cells, only the highest three concentrations reduced % proliferation, and all but the highest concentration reduced the absolute numbers of proliferating cells, again from a very low control level.

**FIGURE 9:**
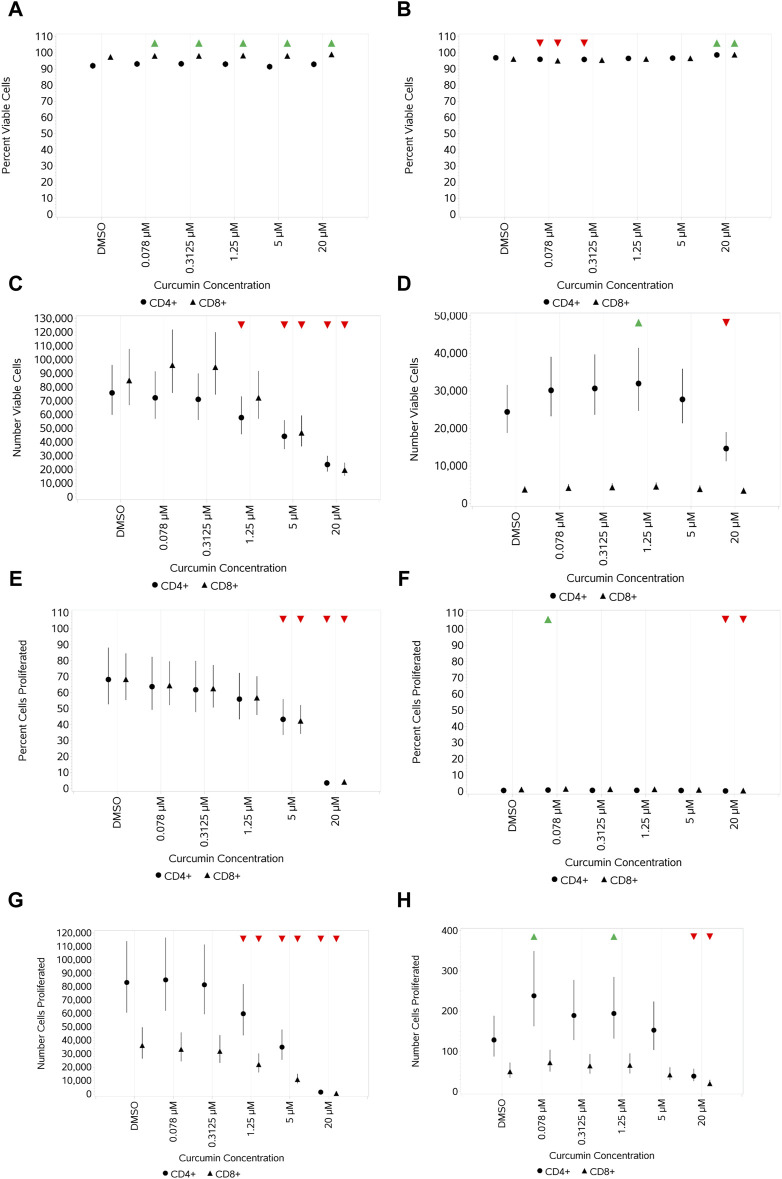
Impact of curcumin on CD4 and CD8 T-cell viability and proliferation after stimulation with either 1.2 μL/mL CS **(A,C,E,G)** or 10 ng/mL HDM **(B,D,F,H)** measured as the percentage viable cells **(A,B)**, absolute number of viable cells **(C,D)**, percentage of proliferating cells **(E,F)**, and number of proliferating cells **(G,H)**. Red triangles denote a significant decrease compared to the unstimulated control, and green triangles indicate a significant increase. Points and lines represent the mean and 95% confidence interval of the mean after the donor effect has been accounted for (eight donors, each with three measurements), respectively.

To illustrate the application of the system for studying the effects of curcumin on T cells, the results of treatment with curcumin on response to stimulation with 1.2 μL/mL CS and 10 ng/mL HDM are given in Figures 9A–H. These two stimuli and doses were chosen for the illustration of application as they had significant, but in some cases different, impacts on the various endpoints measured.

Curcumin treatment resulted in a negligible-to-small increase in percent viable CD4^+^ and CD8^+^ T cells stimulated with CS at 1.2 μL/mL compared to -cells without curcumin treatment (Figure 9A), whereas a concentration-dependent decrease in the absolute numbers of viable CD4^+^ and CD8^+^ T cells, which were similar, was observed at the top 2–3 concentrations of curcumin (Figure 9C). In contrast, curcumin treatment led to a small increase in percent viable CD4^+^ and CD8^+^ T cells only at the top concentration of 20 µM when stimulated with HDM (Figure 9B), and only absolute numbers of CD4^+^ T cells were impacted by curcumin, which showed a concentration-dependent decrease at 5 and 20 μM, reaching a significance level at 20 µM (Figure 9D). The impact of curcumin on proliferation mirrored that on the absolute viable cell number for CS-stimulated cells with a concentration-dependent reduction in both % and absolute number of proliferating cells at the top 2–3 concentrations. Percentage proliferation was low overall with HDM, and curcumin only had a small impact at the highest concentration of curcumin (Figure 9F). Absolute numbers of proliferating CD4^+^ T cells were higher than that of CD8^+^ T cells, and only the top concentration of curcumin had a significant impact (reduction) on both (Figures 9G, H for CS- and HDM-stimulated cells, respectively).

#### 3.4.2 Impact of curcumin on T-cell activation markers CD25 and CD71 unstimulated and stimulated with CS or HDM

**FIGURE 10:**
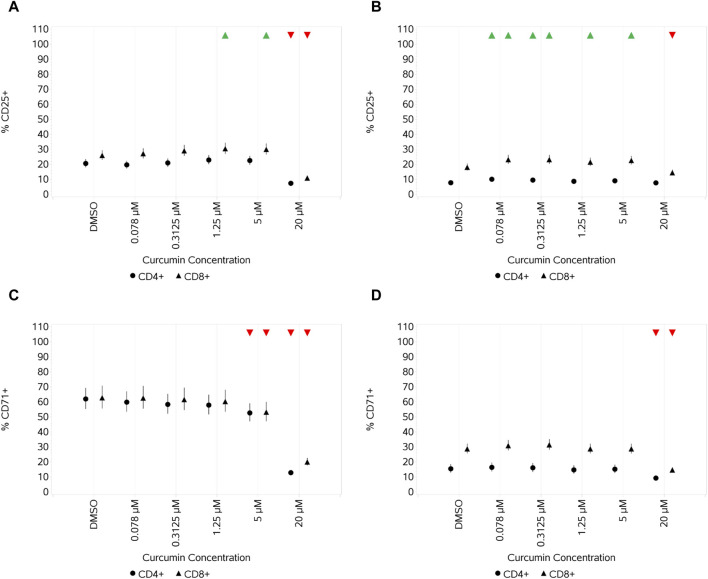
Impact of curcumin on the activation markers CD25 and CD71 on CD4 and CD8 T cells after stimulation with either 1.2 μL/mL CS **(A,C)** or 10 ng/mL HDM **(B,D)** measured as % CD25^+^ cells **(A,B)** and % CD71^+^ cells **(C,D)**. Red triangles denote a significant decrease compared to the unstimulated control, and green triangles indicate a significant increase. Points and lines represent the mean and 95% confidence interval of the mean after the donor effect has been accounted for (eight donors, each with three measurements), respectively.

No significant impact was observed across the curcumin concentration range on the percentage of CD25- and CD71-expressing unstimulated CD4^+^ and CD8^+^ T cells (data not shown), except for a small increase in the percentage of CD25-expressing CD8^+^ T cells with 20 µM curcumin and a corresponding small decrease in the percentage of CD71-expressing CD8^+^ T cells. CS stimulation resulted in a small decrease in the percentage of CD25-expressing CD4^+^ and CD8^+^ T cells only at the highest concentration of curcumin (Figure 10A). With HDM, this effect was observed only in CD8^+^ T cells (Figure 10B). A decrease in the percentage of CD71-expressing CD4^+^ and CD8^+^ T cells stimulated with CS was observed at the top two concentrations of curcumin, but when stimulated with HDM, this was only observed at the highest concentration (20 µM) (Figures 10C, D).

#### 3.4.3 Impact of curcumin on cytokine production by PBMCs unstimulated or stimulated with CS or HDM

When unstimulated PBMCs (data not shown) were treated with curcumin, a general even suppression (no concentration response) in cytokine production was observed across all concentrations of curcumin for IL-2 and IL-6. For IFNγ, TNFα, IL-10, and IL-8, a general pattern of suppression that lessened from 0.078 to 5 μM was observed before decreasing again significantly for all at 20 µM. No impact of curcumin was observed at any concentration on IL-17A.

**FIGURE 11:**
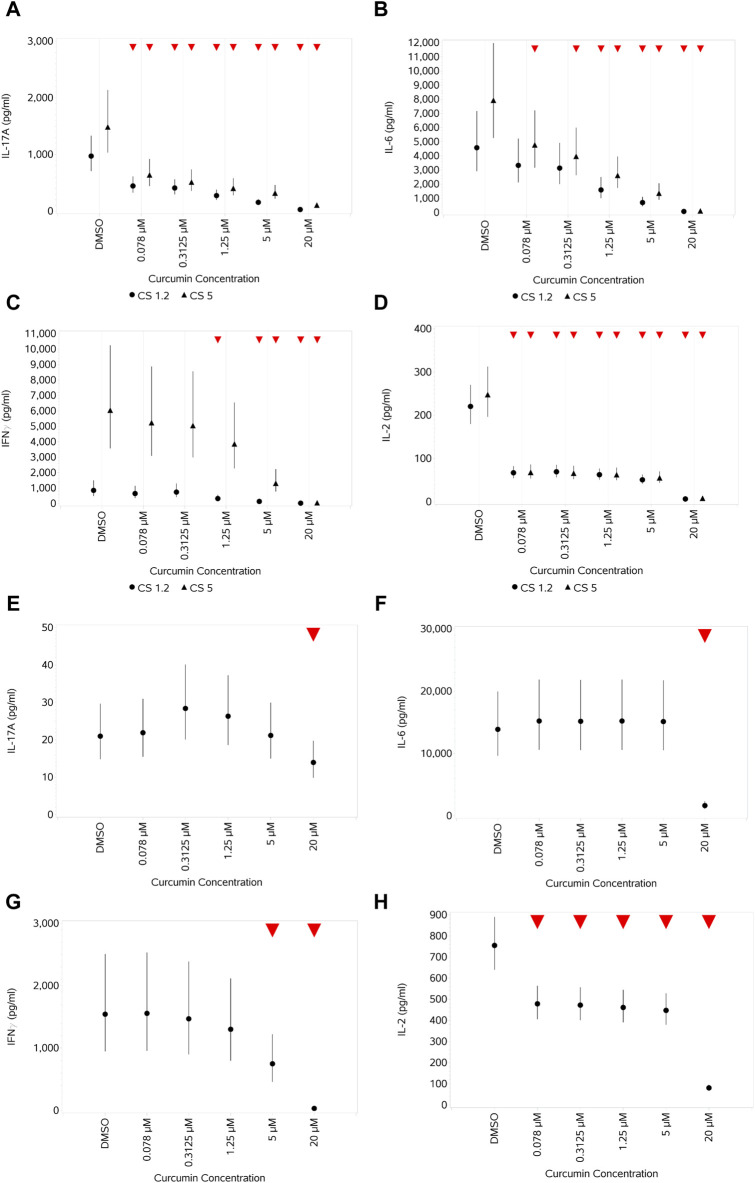
Impact of curcumin on the levels of a selection of cytokines produced by CD4^+^ and CD8^+^ T cells after stimulation with either 1.2 or 5 μL/mL CS **(A–D)** or 10 ng/mL HDM **(E–H)**: IL-17A **(A,E)**, IL-6 **(B,F)**, IFNγ **(C,G)**, and IL-2 **(D,H)**. Red triangles denote a significant decrease compared to the unstimulated control, and green triangles indicate a significant increase. Points and lines represent the mean and 95% confidence interval of the mean after the donor effect has been accounted for (eight donors, each with three measurements), respectively.

When CS-stimulated cells were treated with curcumin, a concentration-dependent suppression of IL-17A, IL-6, and IL-10 across all the concentrations of curcumin could be observed when stimulating with either 1.2 or 5 μL/mL CS (figures provided for IL-17A and IL-6 (Figures 11A, B)). For IFNγ (Figure 11C), suppression was observed only at higher concentrations and was more discernible, although with increased variability, when cells were stimulated with the highest CS concentration. For IL-2 (Figure 11D), a relatively even suppression across the first four concentrations of curcumin was observed before a further decrease at the highest concentration. Against a background of very high expression levels of IL-8, only the highest concentration of curcumin resulted in a decrease in expression (and then only small) in stimulated cells (data not shown). Of note, the result of HDM stimulation was weaker than that of CS, IL-17A, and IL-6 (Figures 11E, F) and was only suppressed at the highest curcumin concentration in HDM-stimulated cells. IFNγ (Figure 11G) levels decreased in a dose-dependent pattern at the higher curcumin concentrations, reaching a significance level at the two highest concentrations. IL-2 showed a similar pattern of response to curcumin treatment (Figure 11H) as observed with CS-stimulated cells.

#### 3.4.4 Impact of curcumin on B-cell viability, proliferation, and activation markers unstimulated and stimulated with CpG or PWM (data not shown)

When unstimulated PBMCs (data not shown) were treated with a range of concentrations of curcumin (0.078 µM–20 µM), no significant impact on the percentage of viable B cells was observed at the lowest three concentrations, but a small concentration-dependent increase was observed at the highest two concentrations. No concentration-dependent change in the absolute numbers of viable B cells was observed. No concentration-dependent impact on the percentage of proliferating B cells was observed, but there was some suppression of the absolute numbers of proliferating B cells across all concentrations.

On CpG stimulation, only a small decrease (<10%) in the percentage of viable cells was observed at the two highest curcumin concentrations. A similar decrease in the absolute number of viable B cells was observed only with the highest curcumin concentration. No significant impact at any concentration of curcumin was observed on PWM stimulation with respect to the percentage of viable B cells, but a small and significant increase in the absolute number of viable B cells was observed with 0.078–5 µM curcumin. A similar pattern was observed for proliferation.

In terms of activation markers, the treatment of unstimulated B cells led to no or small non-concentration-dependent decreases at lower concentrations of all, with a significant increase in the expression of CD71 and CD86 at 20 μM, most marked for CD86. With CpG stimulation, no significant change was observed with curcumin treatment in CD71 expression, a small increase in HLA-DR expression was observed across all curcumin concentrations, and a small increase was observed only at 20 µM for CD86 expression. In contrast, with PWM, no change was observed in CD86 expression, and for CD71 and HLA-DR, the only change was a decrease with the highest curcumin concentration.

#### 3.4.5 Impact of curcumin on monocyte viability unstimulated and stimulated with Pep or LPS

**FIGURE 12:**
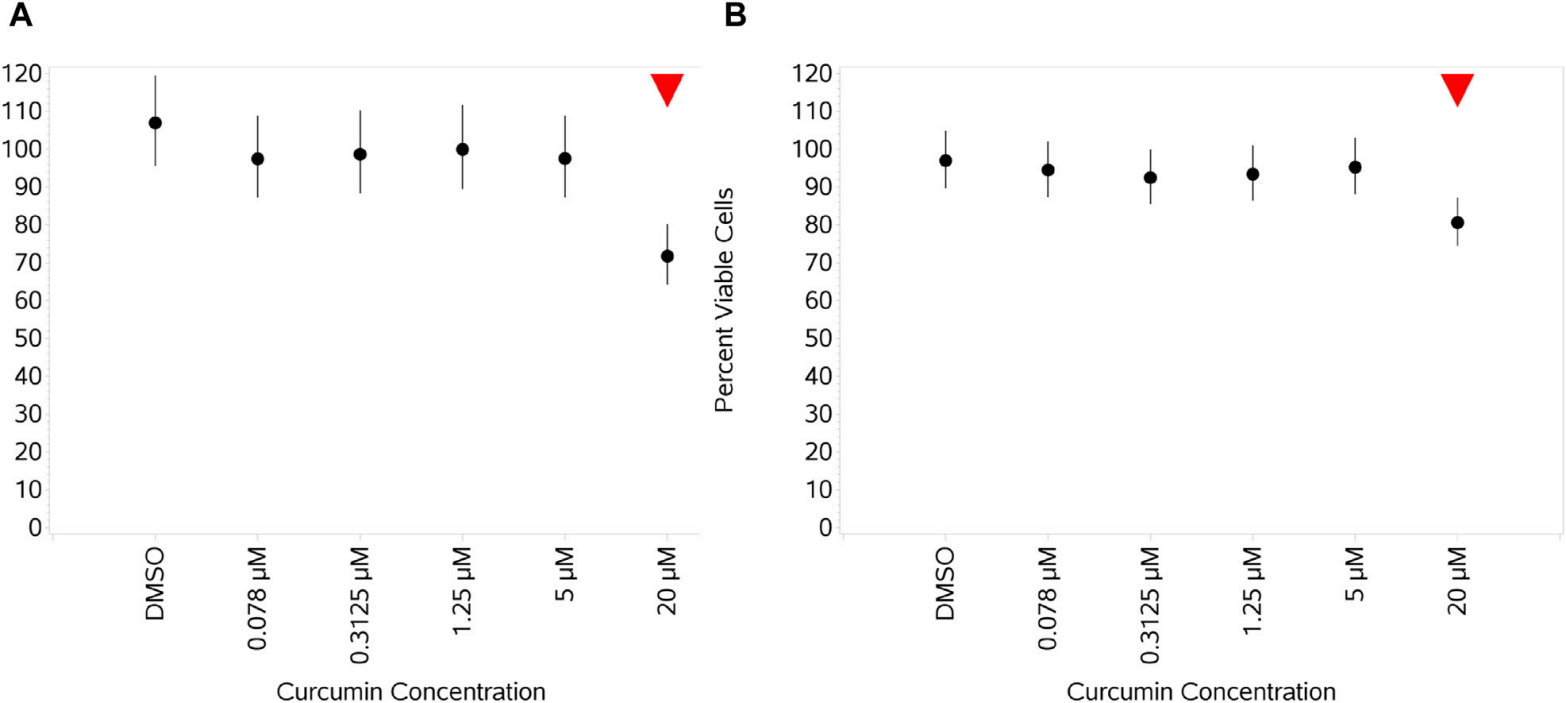
Impact of curcumin monocyte viability and proliferation after stimulation with either 1 ng/mL LPS **(A)** or 100 ng/mL Pep **(B)** measured as percentage viable cells. Red triangles denote a significant decrease compared to the unstimulated control, and green triangles indicate a significant increase. Points and lines represent the mean and 95% confidence interval of the mean after the donor effect has been accounted for (eight donors, each with three measurements), respectively.

Curcumin had no impact on the absolute number of viable monocytes when unstimulated, and the only impact observed on the percentage viability of monocytes was a small reduction at 20 µM (data not shown). With stimulation, the impact was the same, i.e., just a reduction at the highest concentration (Figures 12A, B for LPS and Pep, respectively). No impact/no concentration-dependent impact was observed on the absolute numbers of monocytes with stimulation.

#### 3.4.6 Impact of curcumin on monocyte activation markers CD25, CD86, and HLA-DR unstimulated and stimulated with Pep or LPS

**FIGURE 13:**
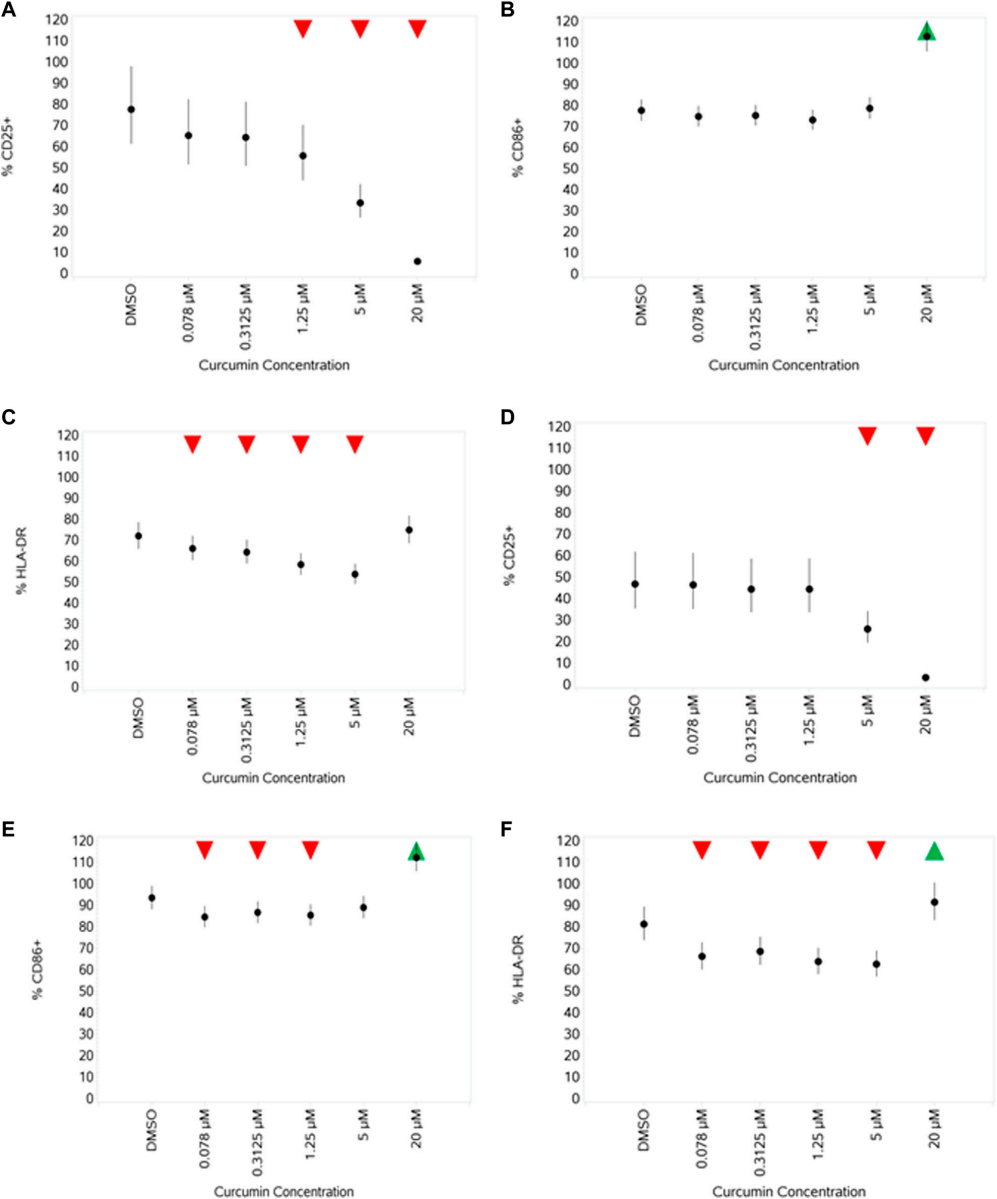
Impact of curcumin on the activation markers CD25, CD86, and HLA-DR on monocytes after stimulation with either 1 ng/mL LPS **(A–C)** or 100 ng/mL Pep **(D–F)** measured as % CD25^+^ cells **(A,D)**, % CD86^+^ cells **(B,E)**, and % HLA-DR^+^ cells **(C,F)**. Red triangles denote a significant decrease compared to the unstimulated control, and green triangles indicate a significant increase. Points and lines represent the mean and 95% confidence interval of the mean after the donor effect has been accounted for (eight donors, each with three measurements), respectively.

In unstimulated cells (data not shown), curcumin led to a very small but significant decrease in CD25 expression at the highest concentration, an increase in CD86 expression also at the highest concentration, and a reduction in HLA-DR across all concentrations, with no obvious concentration-dependent pattern. With both stimuli (Figures 13A–F, A–C, LPS and D–F, Pep), there was a concentration-dependent decrease in CD25 expression (significant at the top 2–3 concentrations), an increase in CD86 expression at the highest concentration, and suppression of HLA-DR expression at all but the highest curcumin concentrations, which instead increased, with this increase reaching a significance level with Pep (Figure 13F).

#### 3.4.7 Impact of curcumin on cytokine and PGE2 production by PBMCs unstimulated or stimulated with Pep or LPS

**FIGURE 14:**
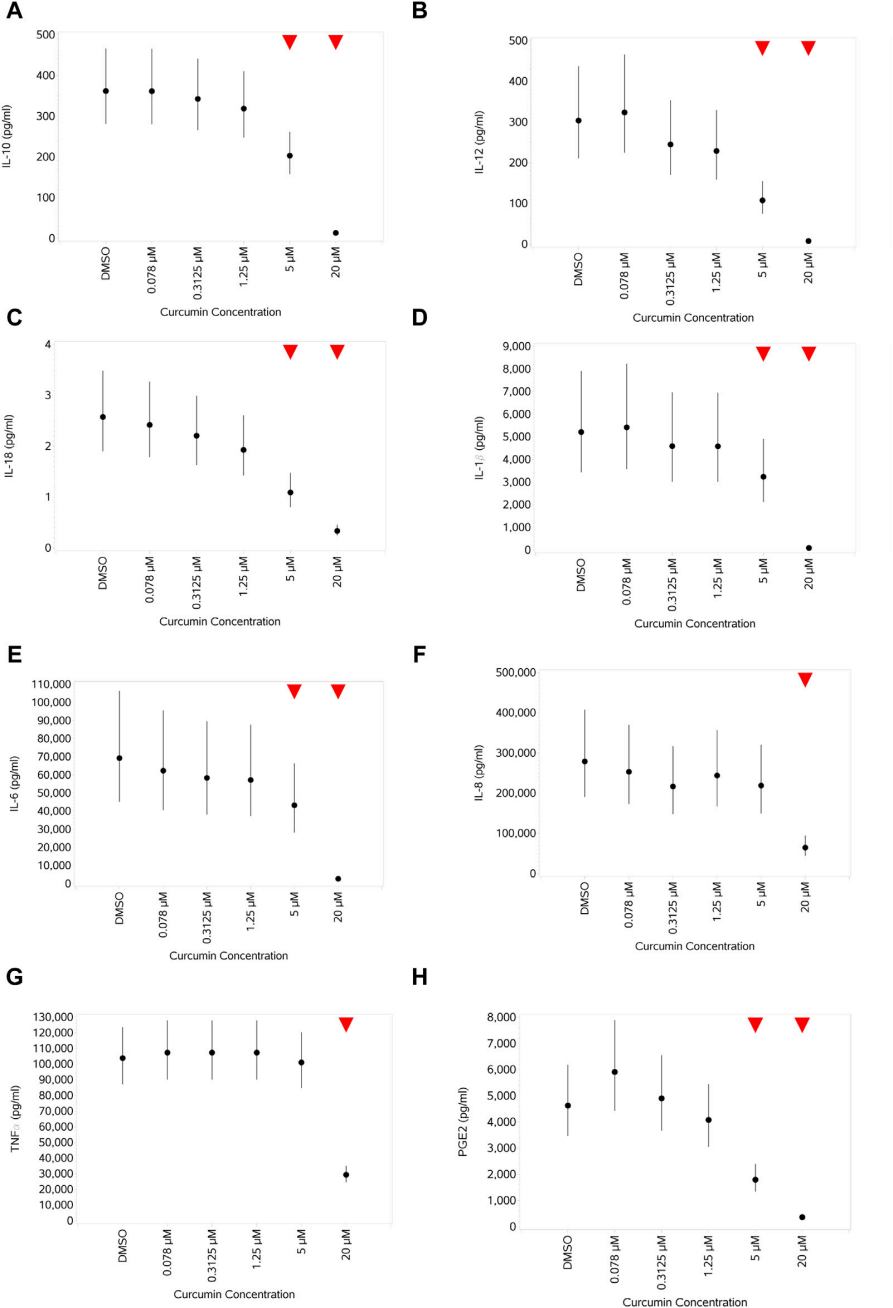
Impact of curcumin on levels of a range of cytokines and PGE2 produced by monocytes after stimulation with 1 ng/mL LPS: IL-10 **(A)**, IL-12 **(B)**, IL-18 **(C)**, IL-1β **(D)**, IL-6 **(E)**, IL-8 **(F)**, TNFα **(G)**, and PGE2 **(H)**. Red triangles denote a significant decrease compared to the unstimulated control, and green triangles indicate a significant increase. Points and lines represent the mean and 95% confidence interval of the mean after the donor effect has been accounted for (eight donors, each with three measurements), respectively.

In unstimulated cells, curcumin had a variable impact on cytokine expression (data not shown). IL-12 was not impacted at all, whereas for all others, an increase at the lower 2–4 concentrations was observed, followed by a decrease that was not significant in the cases of IL-10, IL-18, and IL-8 but was significant at the highest curcumin concentration for TNFα, IL-1β, and IL-6. PGE2 was barely impacted, with only a small increase at 0.3125 µM. With both stimuli, this pattern changed to a concentration-dependent decrease in the levels of all cytokines and of PGE2 at the higher curcumin concentrations, reaching a significance level at the top 1–2 concentrations. The impact on the expression of cells stimulated with LPS (1 ng/mL) and treated with curcumin is shown in Figures 14A–H.

## 4 Discussion

This work is part of a wider effort to develop and apply next-generation risk assessment approaches for the safety assessment of materials (Carmichael et al., 2022). As mentioned in the introduction, the assessment of potential adverse effects on the immune system is an important component of the safety assessment of materials and one of increasing regulatory focus, but the complexity and diverse distribution of the immune system pose multiple challenges in this respect (De Castelbajac et al., 2023; Marx-Stoelting et al., 2023). Several assays have been described for the *in vitro* investigation of immunomodulatory effects (Maddalon et al., 2023) both in single-cell types or co-cultures considering other niche cellular environmental factors, and some of these are also commercially available [e.g., the BioMAP Diversity PLUS assay for the profiling of compounds using 12 human primary cell-based co-culture systems (Simms et al., 2021)]. Depending on the immune cell type or responses of interest, specific assays can be integrated into a battery of tests that can further be used in a tiered manner (Dent et al., 2018; Carmichael et al., 2022).

For the *ab initio* investigation of potential immunomodulatory effects, a human PBMC-based assay system was chosen for development as PBMCs (i) can be easily obtained from blood samples; (ii) have direct relevance as they are derived from humans; and (iii) provide coverage of the major arms of the human immune system through containing the major constituent cell types. This also enables the stimulation and enhancement of specific functional cell subsets and ensures that there is a level of multi-cellular crosstalk, upon which any higher tier testing can be based as per the refinement intended.

A range of stimuli, cell subset activation markers, and cytokines were evaluated and quantified to enable the evaluation of potential immune suppression or stimulation in the safety assessment of materials. The system needs to capture cross-talk between cell types (which may amplify responses) and be amenable to application to large numbers of donors rapidly (as identifying rare donor responses is important in immunotoxicological assessment). The system as described can also provide a steer for follow-on studies if a detailed mechanistic understanding of identified immunomodulation is required. Such studies could include assessments of effects on purified cell populations, the use of additional lineage-specific markers, and intracellular cytokine staining.

### 4.1 T-cell assays

The different stimuli elicited different responses not only in relation to the “quantity” of the response for a given endpoint but also the “quality.” Tetanus toxin, at all concentrations, had a limited impact on all parameters measured. The HDM extract had a mixed impact, and CS had the greatest impact. This, to some degree, is not only as expected, given the mechanisms involved and the expected lower frequency of TT- and HDM-specific T cells, but also illustrates the need to consider using different stimuli. None of the stimuli were “cytotoxic” in that the percentage viable cell numbers remained high (>80%) across all concentrations, although there was a small reduction. Analyzing the absolute viable cell numbers, a different picture emerges, with an initial increase in viability with the lowest CS concentration, tipping to a significant decrease with the highest concentration. All three stimuli increased the proliferation of both T-cell types, although CS was much stronger than TT and HDM, with the lowest CS concentration driving the greatest proliferation. The decrease in viability observed despite an increase in proliferation upon stimulation indicates that there may be some underlying cell death impacting the percentage of viable cells, with the trade-off between proliferation and cell death specific to the stimulus, its concentration, and subset of T-cell population.

These findings illustrate the importance of measuring both percentage viability and absolute viable cell numbers, together with equivalent proliferation data to gain an insight into the impact of stimuli and identify activation-induced death.

At the time point studied, the baseline expression of both CD25 and CD71 was low, as expected without activation. Stimulation with TT and the HDM extract did increase CD25 expression, but the level of increase as measured by both the percentage of activated cells and MFI was much lower than that induced by the two lower concentrations of CS. It was also noted that HDM impacted CD8^+^ T-cell expression of these markers more than CD4^+^ T cells. CS drove a greater increase at the two lower concentrations, with the impact decreasing at the highest concentration, indicating that the highest concentration was over-stimulating. CD71 expression was noted to be more variable for CD8^+^ T-cell MFI.

While the origin of the cytokines in these assays cannot be assigned, the test stimuli differed in the magnitude and type of response. TT had a limited impact on all measured samples. Both CS and HDM promoted the production of several of them but with some notable differences. CS, for example, was the only stimulus to affect IL-17A production and induced greater levels of IFNγ than HDM. In contrast, HDM induced greater levels of IL-2, IL-6, and TNFα, although with higher level-associated variation. For some concentrations of stimuli, cytokine responses were not maximal, but for others, they enabled the potential to look for immune enhancement at lower concentrations and suppression at higher concentrations. Additionally, comparison of stimuli enables the evaluation of a “selective” response initiated by a fraction of presumably antigen-specific T cells vs a polyclonal response to CS likely to activate the majority of CD4 and CD8 T-cell populations.

Using this assay system, a range of effects on CD4 and CD8 T cells could be discerned at different concentrations of curcumin and for the different types of T-cell stimuli, producing an overall picture of a concentration-dependent suppression of activation and cytokine expression at higher concentrations, reflecting an impact on cell viability, such as has been reported by Kliem et al. (2012); Kim et al. (2013). This aligns with the inhibitory effects of curcumin on T-lymphocyte proliferation reported by Deters et al. (2008). Additionally, when the impact of curcumin on the number of proliferating cells is considered, significant inhibitory effects were observed at a similar concentration to that used by Deters et al., who reported that an IC_50_ value of 2.8 µM curcumin for OKT-3 induced PBMC proliferation. It also aligns with the findings of Forward et al. (2011), who reported the inhibition of IL-2 at similar concentrations of curcumin (5–20 µM) and CD25 expression by mouse CD4^+^ T cells in response to antibody-mediated cross-linking of CD3 and CD28. Oh et al. (2018) also reported the regulation of IL-2 by curcumin, reporting direct binding of curcumin to IL-2 in the micromolar range.

### 4.2 B-cell assays

Both stimuli increased the percentage number of viable B cells. However, while this was also the case for the total number of viable B cells with CpG, PWM failed to increase the absolute number of viable B cells compared to unstimulated cells. Given that both stimuli significantly increased the percentage and number of proliferating B cells, this likely reflects the T-cell-independent and T-cell-dependent modes of action of CpG and PWM, respectively.

Both stimuli also significantly increased the expression of all three activation markers (both percentage activated and MFI) compared to unstimulated cells, although PWM was a stronger stimulus of CD71 and CD86. Noting that the baseline expression levels of HLA-DR were higher than that of CD71 and CD86, the increase in the percentage activated cells was equivalent for the two stimuli, although PWM induced a higher MFI than CpG.

In addition to their role in antibody production and ability to produce cytokines, B cells are important antigen-presenting cells (APCs), particularly in secondary (memory) responses. The constitutive high-level MHC class II expression observed attests to this, and the activation-induced expression of co-stimulatory molecules provides an indicator for the APC capacity of B cells.

The consistently greater cytokine response evident with PWM over CpG observed was in keeping with this mitogen, engaging multiple cell types within the PBMC sample. Cross-talk between B- and T-cell populations may also be important in driving T-cell activation and intracellular cytokine staining of surface phenotyped cells, or depletion of B cells could be performed to investigate the direct contribution of different cell types to the different cytokine responses. Some cytokines, such as IFNγ, can exert potent inhibitory effects on B-cell responses (e.g., Reynolds et al., 1987; Abed et al., 1994), and so, the impact of the test compounds on other aspects of the B-cell response should be considered in the context of this.

Curcumin had a limited impact on the B-cell parameters measured with the stimuli used, but it was noted that in contrast to T cells, where there was either no impact or a reduction in viability and proliferation with increasing concentrations, for B cells, a small but significant increase in both was observed at concentrations below 20 µM. Similarly, while the expression of activation markers by T cells was reduced by the highest concentration of curcumin, it was increased in B cells.

### 4.3 Monocyte assays

Cells of monocytic lineage play a critical role in innate immunity, and good responses to both Gram-negative (LPS) and Gram-positive (peptidoglycan) bacterial components were observed. The concentrations of LPS did not impact the percentage number of viable cells, but a significant decrease in viability upon stimulation by peptidoglycan was observed at 1,000 ng/mL.

Effects on CD86 and HLA-DR on monocytes, upon stimulation, were opposite to what was observed with B cells, where stimuli resulted in an increase in CD86 and HLA-DR expression (both % activated cells and the MFI). However, unlike with B cells, the baseline number of activated monocytes expressing CD86 was much higher and the MFI was equivalent, while the % activated monocytes expressing HLA-DR was similar and the MFI was much lower. The reduction in monocyte CD86 surface expression on stimulation with peptidoglycan and LPS is, however, consistent with a previous report and may mechanistically resemble endotoxin tolerance (Wolk et al., 2000).

Stimulation with either Pep or LPS evoked an increase in monocyte CD25 expression, consistent with the other studies of activation-induced expression on monocytes (Scheibenbogen et al., 1992; Farina et al., 2004). Jørgensen et al. showed no effect on CD86 surface expression upon stimulation of CD14^+^ monocytes, from PBMCs, when stimulated with Pep (10 μg/mL) or LPS (10 ng/mL) for 6 h (Jorgensen et al., 2001). Jørgensen et al. also showed an increase in HLA-DR surface expression when monocytes were stimulated with either Pep (10 μg/mL) or LPS (10 ng/mL) for the same shorter duration of 6 h. It is contrary to what was observed in this study, with monocytes stimulated for 24 h with Pep (up to 1 μg/mL) or LPS (up to 100 ng/mL). However, a decrease in HLA-DR on monocytes has been reported in other instances, such as in patients with sepsis with compensatory anti-inflammatory response syndrome (CARS) (Haveman et al., 1999).

The finding of an increase in PGE2 expression upon stimulation by LPS or Pep is consistent with other reports where the stimulation of PBMCs with either of the bacterial cell wall components induced increased PGE2 production (Payvandi et al., 2004; Villamon et al., 2005; Bryn et al., 2008). This is consistent with the LPS-induced activation of TLR4 (Hernandez et al., 2011) and published reports of LPS stimulation in PBMCs (Bryn et al., 2008). Stimulation with peptidoglycan, thought to signal through TLR2 and NOD-like receptors (Mercier et al., 2012; Selvanantham et al., 2013; Chandler and Ernst, 2017), also evoked an increase in PGE2 levels. The increase in the levels of IL-1β, IL-6, IL-8, IL-10, IL-18, and TNFα evoked by both peptidoglycan and LPS is consistent with their well-established role as inflammatory stimuli. LPS regulates the production of cytokines, such as TNFα, the IL-1 family, IL-6, IL-8, the IL-10 family, the IL-12 family, and IL-15, in human monocytes and macrophages (Rossol et al., 2011). Some of the previously reported key pro-inflammatory mediators activated in human monocytes by Pep include IL-1β, IL-6, IL-8, TNF-α, and IL-10 (Schrijver et al., 1999; Wang J. E. et al., 2000; Wang Z. M. et al., 2000). There are mixed reports in the literature of the selective activation of IL-12 by either Gram-positive or Gram-negative bacteria. Gram-positive bacteria are potent inducers of IL-12 in monocytes compared to Gram-negative bacterial strains (Hessle et al., 2000). Smits et al. reported that Gram-negative commensal bacteria primed human dendritic cells for enhanced Th1 development in an IL-12-dependent manner (Smits et al., 2004), which could be why IL-12 expression was greater with LPS stimulation than peptidoglycan in this assay. This finding is also consistent with a previous report describing the differential role of TLR2 and TLR4 signaling on IL-12 induction in human dendritic cells (Re and Strominger, 2001).

Curcumin only impacted the viability (some reduction) of stimulated monocytes at the highest concentration (20 µM) and had different impacts on different activation markers, reducing CD25 at the higher concentrations while increasing CD86 at the higher concentration. HLA-DR was slightly reduced at lower concentrations before rebounding/increasing at the higher concentration. The level of all cytokines and PGE2 was reduced at higher concentrations, indicative of potential immune suppressive/anti-inflammatory effects. While the anti-inflammatory effect of curcumin is surrounded by a degree of controversy (Nelson et al., 2017), the inhibition of multiple cytokines and PGE2 was evident over multiple donors and is consistent with the findings of many others (e.g., Hidaka et al., 2002; Fahey et al., 2007; Koeberle et al., 2009; Ghandadi and Sahebkar, 2017). The reduction in the magnitude of the stimulation-evoked increase in CD25 expression by curcumin is also consistent with the reported anti-inflammatory effects of this compound previously described (Hewlings and Kalman, 2017).

It was also noted that one donor exhibited an increase in the levels of multiple cytokines in the presence of curcumin in the absence of stimulation. Although curcumin has broad anti-inflammatory properties (including its inhibitory impact evident on multiple readouts in this study), others have reported that it can act as a contact allergen in a dermatological setting (Chaudhari et al., 2015), and this result highlights the value of testing multiple donors to potentially identify low-frequency sensitive individuals.

### 4.4 Assay platform advantages and limitations

The assay platform described herein enables the assessment of healthy baseline monocyte, T-, and B-cell responses and scrutiny of the impact of different stimuli to detect potential for immune suppression or enhancement from exogenous materials. The application of a range of stimuli to the same starting sample and potential for multiple readouts is highly modifiable and provides a holistic, integrated picture of effects covering some key characteristics of immunotoxicants (Maddalon et al., 2023), additionally having the capability for potency “benchmarking” if suitable comparator materials are used in the same study. Indeed, the platform has been used to assess the impact of a range of materials with known anti-inflammatory activity, including curcumin, and the data generated for a larger group of test materials will be the subject of a companion paper illustrating the ability of the platform to identify materials impacting the immune system via different mechanisms and with different potencies and how this correlates with human clinical and exposure data.

As part of the drive to maximize the reliability and human relevance of *in vitro* methods, the impact of the inclusion of animal-derived materials in culture systems has come under scrutiny and is of particular concern in studies of human immune responses due to the immunogenic potential of exogenic materials (Witzeneder et al., 2013). The use of human serum in *in vitro* culture systems addresses the multiple disadvantages associated with the use of fetal bovine serum (van der Valk et al., 2018; van der Valk, 2022) and further enhances human translatability. The use of human serum did, however, come with some challenges. Most notable was the presence of pre-existing human solutes that could potentially confound subsequent measurement in culture media. In the present study, a high basal level of IL-8 was encountered, but despite this, statistically significant increases above this background could be measured. Not shown here, however, was an inability to measure induced levels of immunoglobulins with the two B-cell stimuli used due to high background levels in human serum. To remedy this, chemically defined serum-free cultures were established, the results of which will be the subject of a future study.

The limitations/challenges associated with the platform described are as follows:

1. The assay system described here only captures a single “snapshot” in time, and although three majority subsets of functional immune cells present in PBMCs have been analyzed, it is not a complete subset, and there is no co-culture with other tissues. As already stated, however, the platform is modifiable, and additional targets, stimuli, and readouts can all be added at the outset. For example, the incorporation and evaluation of other key cytokines associated with specific cell subsets may be required, such as the measurement of IL-4, IL-5, and IL-13, which are characteristic of Th2 cells and important in allergic responses (Lambrecht et al., 2019). This may, however, require the enrichment of responding cell types and/or more specialized culture conditions. For reasons of cost and resources, taking a step with an evidence-led tiered approach starting with the system as described in this paper may, however, be most appropriate for the *ab initio* assessment of the potential immunomodulatory effects of test materials for which there are no pre-existing data.
2. Whether there is inherent metabolic capacity for the activation or detoxification of chemical compounds also needs to be considered. While some metabolic pathways have been characterized in PBMCs (Liptrott et al., 2008; Liptrott et al., 2009), there is limited information about the metabolic capacity of PBMCs in relation to *in vitro* chemical exposure. It is for this reason, i.e., to avoid any interference with PBMC responses to different test materials, some of which may act via oxidative stress, that we did not add 2-mercaptoethanol (2-ME) in the culture media. Sometimes, 2-ME is added to lymphocyte cultures to enhance responses, as was found to be the case for murine cells. The impact on human cells is, however, variable and dependent upon the cell type and culture conditions, with greater effects observed in serum-free media (Click, 2014).
3. Although there are undoubted benefits to using primary human PBMCs, such as human relevance/translation and being a relatively easily accessible mixed immune cell population, thus enabling the study of some cross-talk and specific functional subsets of cells, we do recognize that they have associated challenges, such as potential infection risk to laboratory workers handling such samples, although this can be managed, as illustrated by PBMCs being widely used in research and other areas of toxicology. An additional advantage of using primary human PBMCs is the potential to select donors with specific characteristics to enable the assessment of the impact of elements, such as pre-existing conditions, lifestyle factors, such as smoking, or the impact of living in urban and rural areas on responses to test materials.

One “challenge” often cited with such samples is donor-to-donor variation. This “challenge” is, however, also an advantage, providing important data on potential effects at a population level. To utilize such data, some researchers use each donor as their own control, normalizing any percent change from a control response. The statistical analysis undertaken here adjusts for donor variation by fitting a mean for each donor within the modeling process.

For the purposes of screening and guiding further studies, however, this is an accessible platform, amenable to high-throughput (HTP) modifications and highly adaptable, providing a holistic, integrated view of the potential mechanisms of action and relative potency of materials.

As its application will be covered in detail in a future study, only selected curcumin data were provided in this publication. The data provided here do, however, demonstrate how the platform can identify a material with potential anti-inflammatory/immune suppressive effects and generate data that can be taken into a wider framework for consideration in the context of human exposure levels and in relation to comparator materials. Inflammation is a natural response to harmful stimuli, such as infection, and reducing the ability to raise an inflammatory response could lead to adverse outcomes, such as an increased infection risk. Despite there being a plethora of evidence indicative of anti-inflammatory/immune suppressive effects, curcumin has not been marketed as a therapeutic agent despite being well tolerated, with the main limitation to its therapeutic application being poor bioavailability (Peng et al., 2021; Allegra et al., 2022). This illustrates the importance of understanding exposure and *in vitro* to *in vivo* extrapolation when interpreting data from platforms such as that described in this paper. Consideration of different routes of exposure is also important, as although bioavailability may be poor by the oral route, there is also the potential for metabolism via the liver, although this may not apply in the case of direct application to inflamed tissues, e.g., the skin.

Middleton et al. (2022) described a core toolbox and workflow for conducting systemic safety assessments using non-animal approaches for adult consumers that have been developed by Unilever SEAC (Middleton et al., 2022), and the aim is to expand this with other assays to cover an increased range of toxicological endpoints, such as developmental and reproductive toxicology (Rajagopal et al., 2022) and immune modulation (this publication). The toolbox described by Middleton et al. (2022) includes physiologically-based kinetic (PBK) models to estimate systemic levels in humans to provide context to the concentrations of test materials used and implications for safety decision-making.

As mentioned, multiple test materials have been assessed using the platform, and work is underway to further evaluate and consider how to incorporate the assays described into a tiered next-generation risk assessment framework to support animal-free safety decision-making.

## Data Availability

The raw data supporting the conclusion of this article will be made available by the authors, upon reasonable request.
