# Supplementary material for: Impact of a varied set of stimuli on a suite of immunological parameters within peripheral blood mononuclear cells: toward a non-animal approach for assessing immune modulation by materials intended for human use
Source: Front Toxicol. 2024 Apr 26;6:1335110. doi: 10.3389/ftox.2024.1335110 (PMC11082367; doi:10.3389/ftox.2024.1335110)
Supplement: Supplementary file 1 [file Image1.PDF]

Impact of a varied set of stimuli on a suite of immunological parameters within peripheral blood mononuclear cells: towards a non-animal approach for assessing immune modulation by materials intended for human use

An accessible, highly modifiable system that can be used to screen materials and guide further studies, providing a holistic, integrated picture of effects

CONTEXT

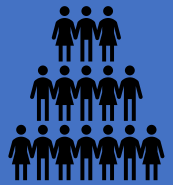

Human Health Risk Assessment

A move to mechanism-focused and human relevant risk assessment requires new approaches and methods

Immunotoxicity is an area of particular need

APPROACH

Peripheral Blood Mononuclear Cells (PBMCs) from 8 donors

↓

Each Donor

↓

PBMCs cultured untreated or with curcumin (0.078-20 µM)

↓ 1h

cultured unstimulated or stimulated for a further 24h (monocyte assays) or 144h (T or B cell assays)

↓

Measurements

Proliferation   Viability   Activation Markers

Cytokines   PGE2

OUTCOME

Assessment of healthy baseline monocyte, T-, and B-cell responses; the impact of different stimuli to detect potential immune suppression / enhancement by exogenous materials

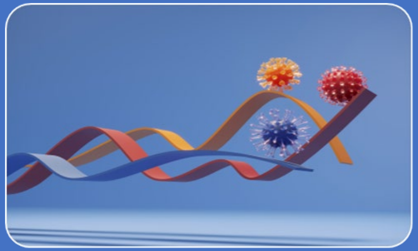

Curcumin: a pattern of responses indicative of immune suppressive / anti-inflammatory effects
